# Study of Polymer Matrix Degradation Behavior in CFRP Short Pulsed Laser Processing

**DOI:** 10.3390/polym8080299

**Published:** 2016-08-15

**Authors:** Hebing Xu, Jun Hu

**Affiliations:** School of Mechanical Engineering, Shanghai Jiao Tong University, Shanghai 200240, China; xuhebing@sjtu.edu.cn

**Keywords:** CFRP, short pulse length, polymer pyrolysis, mechanical erosion

## Abstract

Short pulsed laser is preferred to avoid the thermal damage in processing the heat sensitive material, such as carbon fiber reinforced plastic (CFRP). In this paper, a numerical model capturing both the material ablation and polymer matrix pyrolysis processes in pulsed laser processing is established. The effect of laser pulse length from ns order to μs order is studied. It was found that with shorter pulse length, ablation depth is increased and heat affected zone is remarkably reduced. Moreover the pyrolysis gas transport analysis shows that shorter pulse length results in a larger internal pressure. At pulse length in ns order, maximum pressure as high as hundreds of times atmospheric pressure in CFRP could be produced and leads to mechanical erosion of material. The predicted ablation depth of a single short laser pulse conforms well to the experiment result of the CFRP laser milling experiment.

## 1. Introduction

Advanced composite materials with high specific strength and design-versatility properties are preferred in industries such as aircraft, electric vehicle, sports goods, etc. [1,2]. Among them, carbon fiber reinforced plastic (CFRP) is widely used to manufacture high performance structural components [3]. Although CFRP components are produced in a near-net-shape manner, drilling, cutting, repair, etc. are often conducted on the components to fulfill the assembling and maintenance requirements [4,5].

Owing to the non-contact and high efficiency characteristics, methods of CFRP short pulsed laser processing (ns pulse length, GW/cm^2^ power intensity) have been studied by many researchers [6,7] in recent years to meet the machining requirement of CFRP material which proposes a big challenge to traditional mechanical machining. The experimental results show that the mechanism of material removal and heat affected zone (HAZ) formation where the polymer matrix is damaged in the laser processing is complicated. Ablation, matrix degradation, and mechanical erosion by the recoil pressure coexist in the processing [8]. Under high peak power laser irradiation, carbon fibers and polymer matrix are ablated in a very short time. Meanwhile polymer matrix around the laser-material interaction area is easily pyrolyzed, and the polymer matrix loses its ability to maintain the carbon fibers and transfer the load. Therefore, HAZ becomes the most possible area where structural failure takes place [9]. Herzog et al. found that CFRP’s static strength is inversely proportional to the size of HAZ [10]. The main optimization objects in CFRP laser processing are high machining efficiency and limited HAZ. They obviously rely on material removal mechanism and the processing parameters’ effect. Besides experimental investigations, it is necessary to study the material response under short pulsed laser irradiation through simulation analysis in order to clarify the specific processing characteristics.

As the polymer is much easier to degrade than carbon fiber, many researchers just focus on the direct material ablation in the laser-material interaction area neglecting the polymer matrix degradation process in the numerical model. However, actually the degradation of polymer matrix plays an important role in the heat and mass transport in the CFRP laser processing. When the temperature is high enough, the polymer matrix is pyrolyzed to the char and gas. The gas flow in the material with heat and mass transport through the voids between the char, carbon fiber, and molten polymer matrix. The mass transport yields an internal pressure field which drives the gas outward in return. It was reported that about 20% pressure increase over atmospheric pressure in natural wood could lead to the structural failure [11]. Polymer degradation under common low power intensity heat source, such as fire, has been studied by many researchers [12,13]. They made a model simplification that polymer degrades instantaneously once its temperature exceeds a critical value. Meanwhile the simulation assumed the overall volume is constant neglecting the material surface ablation. However, for CFRP short pulsed laser processing, polymer degradation process should be analyzed with the surface ablation of material. Under the condition that extremely high peak power input generates a large heating rate, the specific polymer degradation and its impact on the whole etching process are not known yet.

In this paper, a numerical model is established to capture both the CFRP short pulsed laser ablation and the formation process of HAZ. In order to accurately predict the polymer pyrolysis in the heating circumstance of short pulsed laser, two polymer degradation models—finite-rate pyrolysis and instantaneous pyrolysis—are compared in order to choose the appropriate one which can be used in the short pulsed laser processing. The pulse duration varies from short pulse length at ns order to long pulse length at μs order to investigate the specific material response. The short pulsed laser milling experiments are performed to validate the numerical model.

## 2. Experimental Setup

**Figure 1:**
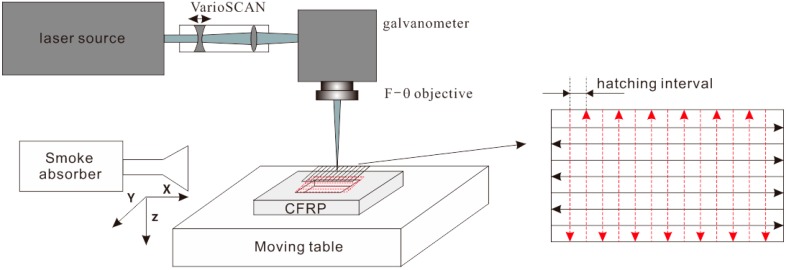
Schematic of short pulsed laser milling of carbon fiber reinforced plastic (CFRP).

The CFRP laser processing experiment is conducted using a 10 ns pulse length, 50 kHz repetition rate, 20W Nd:YVO_4_ laser system as shown in Figure 1. CFRP laminate is mounted on the moving table. The machining surface of CFRP is scanned by the focused laser beam along the *x*-*y* plane through the galvanometer. Meanwhile the focal plane can be adjusted by the VarioSCAN^®^ device (Puchheim, Germany). CFRP used in the experiment consists of TORAYCA^®^ (Tokyo, Japan) T300 carbon fibers, and epoxy matrix. It has two external woven plies and several 0°/90° unidirectional plies inside. The thickness of a single ply is about 0.25 mm and the total thickness of the laminate is 2 mm. Due to the small pulse energy (<1 mJ) and heterogeneity of the material microstructure, ablation depth of a single pulse is very small and inconsistent as well. It is just meaningful to obtain an average value to estimate a single pulse ablation depth. So a laser milling strategy using parallel scanning lines to hatch the whole machining surface is adopted. Through multiple scanning passes, a pocket with a certain depth is produced. The main laser processing parameters and material properties are listed in Table 1.

## 3. Model Formulation

### 3.1. Polymer Degradation Mechanism

**Figure 2:**
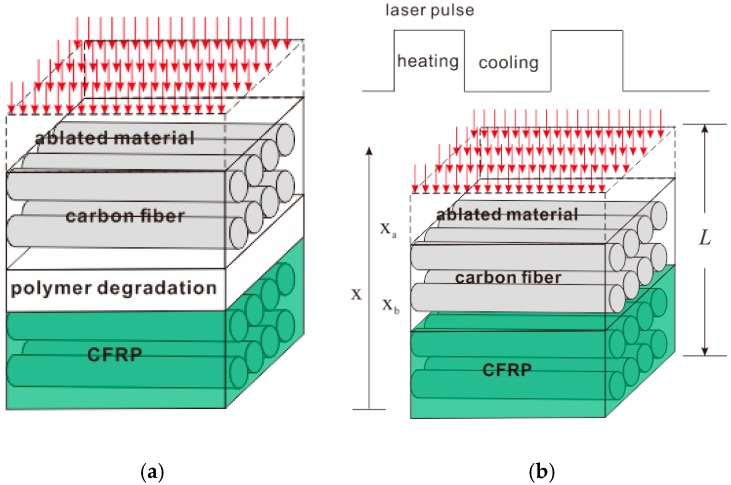
(**a**) Finite-rate pyrolysis model; (**b**) Instantaneous pyrolysis model.

The realistic degradation of polymer is a chemical reaction with a finite rate as shown in the Figure 2a. Therefore in the finite-rate model, polymer degrades in a gradual way. The reaction rate could be calculated by Arrhenius equation [15]. The pyrolysis kinetic parameters including activation energy, pre-exponential factor, and reaction order are determined by thermal gravity analysis (TGA) [16]. In the processing, the material is divided into four layers: CFRP layer, polymer degradation layer, carbon fiber layer, and ablated material layer. In our discussion, it is assumed that polymer just degrades to gas without char generation. In the degradation area layer, polymer partly degrades and the material here is a mixture of gas and molten polymer. In the carbon fiber layer, polymer completely degrades. The voids among the fibers are filled with pyrolysis gas. When the reaction rate is big enough, it can be assumed that the polymer degrades in an instantaneous step once the temperature is above the degradation temperature as shown in Figure 2b. In the instantaneous model, the material just has three layers without the polymer degradation layer. A sharp interface exists between the CFRP layer and carbon fiber layer. This simplified model makes the calculation easier in two aspects. Firstly, the pyrolysis degree just depends on the material temperature and has nothing to do with the time. Meanwhile, due to the instantaneous pyrolysis assumption, there is no polymer partial degradation area where pyrolysis gas and molten polymer coexist making the mass transport modeling very complicated.

In the pulsed mode of laser source, a total cycle contains the single laser pulse heating stage and the cooling stage between two laser pulses. Material ablation and polymer pyrolysis are all dynamic processes resulting in two receding boundaries, ablation boundary $x_{a}$ between the ablated material layer and carbon fiber layer, pyrolysis boundary $x_{b}$ between the carbon fiber layer and CFRP layer. At the heating stage, the two boundaries all move down. At the cooling stage, the ablation stops but polymer pyrolysis continues with the thermal diffusion. Then $x_{a}$ remains unchanged and $x_{b}$ continues to recede. When the polymer pyrolysis completely finishes, $x_{a}$ does not change as well. The ablation depth is ($L-x_{a}$) and the size of HAZ, i.e., carbon fiber layer, is ($x_{a}-x_{b}$).

In the finite-rate pyrolysis model, reaction rate in the pyrolysis is related to the heating rate and temperature. High peak power of short pulsed laser generates a big heating rate and temperature gradient in the material. However, its pyrolysis kinetic parameters could not be obtained by usual TGA experiments which are conducted with slow heating rate 5–60 K/min. A set of pyrolysis kinetic parameters derived from typical TGA is discussed below to show the reasonability of model simplification in short pulsed laser processing. The dynamic pyrolysis is governed by the Arrhenius equation as follows:

(1) $$\frac{dm_{p}(t)}{dt}=-Am_{p}(t_{0}){\left(\frac{m_{p}(t)-m_{p}(t_{\infty })}{m_{p}(t_{0})}\right)}^{n}\exp(-\frac{E_{a}}{RT})$$

where $m_{p}(t)$, $m_{p}(t_{0})$ and $m_{p}(t_{\infty })$ are the current, initial and final mass of the polymer. A(1/s), $E_{a}$ (KJ/mol), and n are the three reaction kinetics: pre-exponential factor, activation energy, and reaction order. For the epoxy, the experimental data are 3.15 × 10^11^, 181.73 and 1.344 which are the average values of three TGA tests in heating rates 5, 10, and 20 K/min in reference [15].

**Figure 3:**
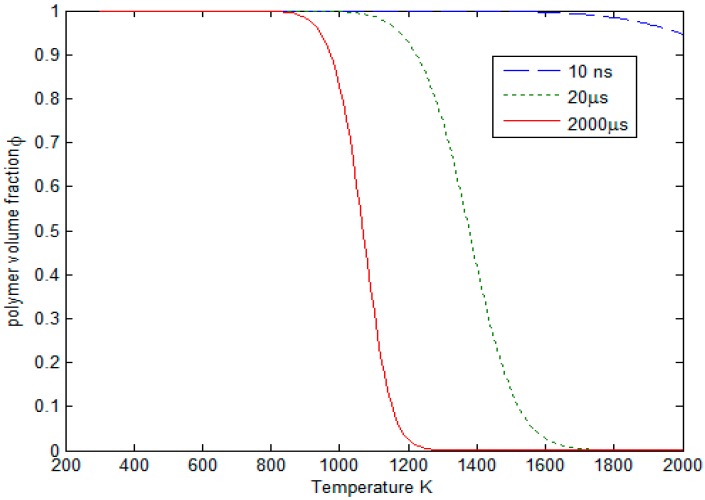
Polymer volume fraction at different temperatures in a certain reaction time (10 ns, 20 μs, 2000 μs).

Using volume fraction ϕ instead of mass in Equation (1), the polymer volume fraction decreases from 1 to 0 as the pyrolysis proceeds. For a certain reaction time, the volume fraction is a function of temperature as shown in Figure 3. When the reaction time increases from 10 ns to 2000 μs, volume fraction curve shifts to the left because more polymer degrades in a longer heating time. It can be seen that when the reaction time is less than 20 μs, there is no obvious polymer degradation at a temperature as high as 1100 K. This is not reasonable because the polymer is already damaged at such a high temperature. In the short pulsed laser processing, temperature surrounding the laser ablation area can easily reach 1000 K less than 20 μs due to the extremely high heating rate. At the cooling stage, the temperature drops quickly by thermal diffusion. Therefore, on this occasion the finite-rate pyrolysis model could not predict the HAZ. The problem comes from the pyrolysis kinetic parameters obtained from the low heating rate TGA test. Actually, the polymer pyrolysis rate is very high at a high laser heating rate. So in the following modeling process, it is assumed that polymer pyrolysis happens in an instantaneous step once the temperature exceeds a critical value so that polymer damage could be considered as much as possible.

### 3.2. Heat Transport

In CFRP laser processing, the heat transport phenomenon contains conduction, convection of pyrolysis gas, carbon fiber sublimation, and polymer matrix pyrolysis. The governing equation is:

(2) $$\rho (\phi_{p}C_{p}+\phi_{f}C_{f}+\phi_{g}C_{g})\frac{\partial T}{\partial t}+\frac{\partial (\rho_{g}\phi vT)}{\partial x}=\frac{\partial }{\partial x}(k\frac{\partial T}{\partial x})-\phi_{p}\rho L_{1}\frac{\partial \alpha_{1}}{\partial t}-\phi_{f}\rho L_{2}\frac{\partial \alpha_{2}}{\partial t}$$

where $\phi_{i}(i=p,\;f,\;g)$, $C_{i}(i=p,\;f,\;g)$ are the volume fraction and specific heat of polymer, carbon fiber and pyrolysis gas, respectively. Material properties of the pyrolysis gas are listed in Table 2. $\alpha_{i}(i=1,\;2)$, $L_{i}(i=1,\;2)$ are the phase change degree and latent heat of polymer and carbon fiber. k is the heat conductivity of CFRP. It is calculated by mixture law using the material properties of carbon fiber and polymer matrix. As the heat mainly conducts along the fibers, the fiber direction is modeled in order to study the HAZ.

In order to eliminate the time dependent latent heat source terms on the right side of Equation (2), change the term $\frac{\partial \alpha_{i}}{\partial t}(i=1,\;2)$ into $\frac{\partial \alpha_{i}}{\partial T}\frac{\partial T}{\partial t}$ , then it is transposed into:

(3) $$\rho C_{eq}\frac{\partial T}{\partial t}+\frac{\partial (\rho_{g}\phi vT)}{\partial x}=\frac{\partial }{\partial x}(k\frac{\partial T}{\partial x})$$

where the equivalent heat capacity $C_{eq}$ is ($\phi_{p}C_{p}$ + $\phi_{f}C_{f}$ + $\phi_{g}C_{g}$ + $\phi_{p}L_{1}\frac{\partial \alpha_{1}}{\partial T}$ + $\phi_{f}L_{2}\frac{\partial \alpha_{2}}{\partial T}$).

In the instantaneous model, $\frac{\partial \alpha_{i}}{\partial T}$ becomes an impulse function which is not permitted. Therefore a temperature interval $\left[T_{1},\;T_{2}\right]$is adopted instead of a critical temperature point. $T_{1}$ and $T_{2}$ are the starting and finishing temperatures of phase change.

At the heating stage, material ablation and matrix pyrolysis proceed at the same time. So the equivalent heat capacity $C_{eq}$ should consider the latent heat as follows:

(4) $$\left\{ \begin{array}{lll} C_{eq}=\phi_{f}C_{f}+\phi_{g}C_{g}+\phi_{f}\rho L_{2}\frac{\partial \alpha_{2}}{\partial T} & & x_{b}\leq x\leq x_{a}\\ C_{eq}=\phi_{p}C_{p}+\phi_{f}C_{f}+\phi_{p}\rho L_{1}\frac{\partial \alpha_{1}}{\partial T}\;+\phi_{f}\rho L_{2}\frac{\partial \alpha_{2}}{\partial T} & & x<x_{b} \end{array}\right.$$

And the boundary condition at $x=x_{a}$ is:

(5) $$-k\frac{\partial T}{\partial x}=A\cdot I_{0}+h\cdot (T_{a}-T)$$

where A is laser absorptivity of CFRP, $I_{0}=\frac{P}{\pi r^{2}}$ is the average power intensity and h is the air convection coefficient.

At the cooling stage, the state of material would not change reversely. Then the $C_{eq}$ is written as:

(6) $$\left\{ \begin{array}{lll} C_{eq}=\phi_{f}C_{f}+\phi_{g}C_{g} & & x_{b}\leq x\leq x_{a}\\ C_{eq}=\phi_{p}C_{p}+\phi_{f}C_{f} & & x<x_{b} \end{array}\right.$$

And the boundary condition at $x=x_{a}$ is:

(7) $$-k\frac{\partial T}{\partial x}=h\cdot (T_{0}-T)$$

At the lower surface x=0, the boundary condition is air convection

(8) $$-k\frac{\partial T}{\partial x}=h\cdot (T_{0}-T)$$

### 3.3. Mass Transport

In the instantaneous model, pyrolysis gas generated in the pyrolysis flows outward through the carbon fiber layer. The porous carbon fiber structure provides resistance to the gas flow resulting an internal pressure field. Conservation of the gas is governed by the equation:

(9) $$\frac{\partial }{\partial t}(\phi \rho_{g})=-\frac{\partial }{\partial x}(\rho_{g}v\phi )\;x_{a}\leq x\leq x_{b}$$

where ϕ is the volume fraction of polymer matrix in CFRP. $\rho_{g}$ is the density of pyrolysis gas. v is the gas filtration speed in the carbon fiber layer. Darcy’s law has been applied in the groundwater flow and oil flow. It is also adopted here to estimate the speed of pyrolysis gas:

(10) $$v=-\frac{\gamma }{\phi \mu }\nabla P$$

where γ is the permeability of the porous medium. μ is the dynamic viscosity of pyrolysis gas. 

In CFRP short pulsed laser processing, the porous medium is the carbon fiber layer which consists of cylindrical carbon fibers. Its permeability is represented by a function of carbon fiber diameter $d_{f}$ (≈7 μm) and gas volume fraction *φ* as follows [15]:

(11) $$\gamma =\frac{d_{f}^{2}\phi^{3}}{180{\left(1-\phi \right)}^{2}}$$

Substituting the gas density by temperature and pressure according to the ideal gas law $\rho_{g}=\frac{P\cdot M_{g}}{T\cdot R}$ in Equation (9), we can obtain:

(12) $$\frac{\partial }{\partial t}(\frac{\hat{P}}{\hat{T}})=\frac{\gamma P_{a}}{\phi \mu }\frac{\partial }{\partial x}(\frac{\hat{P}}{\hat{T}}\frac{\partial \hat{P}}{\partial x})$$

where the dimensionless pressure and temperature ($\hat{P}$, $\hat{T}$) are ($\frac{P}{P_{a}}$, $\frac{T}{T_{a}}$). $P_{a}$, $T_{a}$ are the atmospheric pressure 101.3 kPa and environment temperature 300 K.

The boundary condition at $x_{b}$ is the polymer pyrolysis depicted as:

(13) $$\rho_{g}\frac{\gamma P_{a}}{\mu }\frac{\partial \hat{P}}{\partial x}=\phi (\rho_{p}-\rho_{g})\frac{dx_{b}}{dt}\;x=x_{b}$$

The boundary condition at $x_{a}$ is the atmospheric pressure as follows:

(14) $$\hat{P}=1\;x=x_{a}$$

where $\rho_{p}$ is the density of polymer.

## 4. Results and Discussion

**Figure 4:**
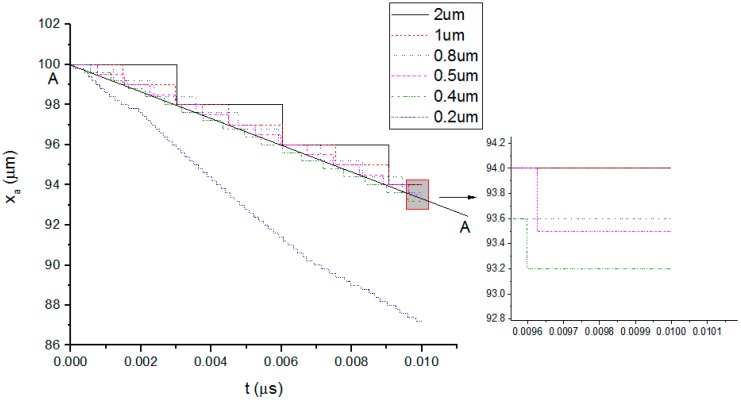
Effect of space step *δx* on the ablation boundary at time step *δt* = 0.001 ns.

The effect of laser pulse length on the polymer pyrolysis behavior is studied in the model. Five different pulse lengths including short length 10 ns, three long lengths (5, 10, 15 μs), and the continuous wave 20 μs as the repetition rate is 50 kHz. Other laser processing parameters and material properties are listed in Table 1. Firstly the effect of space step δx at short length 10 ns which has the biggest peak power is analyzed. Figure 4 shows the curves of $x_{a}$ at time step δt=0.001 ns and the whole modeling range L is 100 μm. “Birth-death element” method is used to model the material removal. Thermal conductivity of the element whose temperature is over 3900 K is set to be zero. Meanwhile, ablation boundary $x_{a}$ recedes by δx at a time. In the situation that the space step δ*x* is too bigger, discrete effect of the model, i.e., step shape of the curve, is very obvious. However, when *δx* is too small, the time step δt must be decreased to guarantee the convergence of the simulation which brings large calculation. The predicted ablation depth deceases with smaller space step. It can be seen that at space step (δ*x* = 0.5~2 μm), the curves have the same envelop curve A–A indicating that the material is linearly ablated under such great peak power. As the heat diffusion is small compared to the absorbed laser energy, ablation boundary $x_{a}$ recedes steadily at constant laser power. When the space step is further decreased to 0.4 and 0.2 μm, the curves begin to deviate from the A–A line. The time step in these situations should be decreased to improve the simulation accuracy. Therefore, in the following model, the space step is set to be 0.5 μm and there are 200 elements in total.

### 4.1. Characteristics of 10 ns Short Pulsed Laser

**Figure 5:**
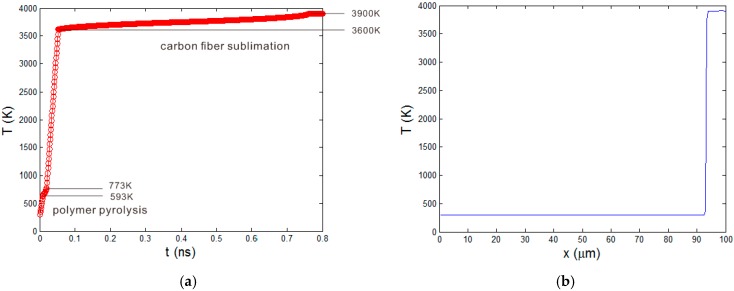
(**a**) Temperature curve at *x* = 100 μm; (**b**) Temperature field at 10 ns.

Figure 5a shows the temperature curve at *x* = 100 μm until it reaches the ablation temperature of carbon fiber. The curve can be divided into five zones. (i) T = 300~593 K. The material is the original CFRP layer; (ii) T = 593~773 K. Polymer pyrolysis starts. The slope of temperature curve becomes small as the latent heat of polymer pyrolysis is considered in the equivalent heat capacity $C_{eq}$; (iii) T = 773~3600 K. The temperature rises quickly as the polymer matrix completely degrades; (iv) T = 3600~3900 K. Carbon fibers are gradually removed by sublimation. The temperature rising rate is the smallest as the phase change latent heat of carbon fiber is very big; (v) T = 3900 K. The element is killed and the temperature is kept unchanged. The temperature field at the end of the short laser pulse is shown in Figure 5b. In the curve, the place where the temperature is above 3900 K is the ablated material. As the heating rate is very high, there exists a great temperature gradient around the ablation boundary. The temperate drops from the ablation temperature almost directly to the initial ambient temperature. This makes the two boundaries $x_{a}$ and $x_{b}$ coincide together at the heating stage of the short pulse length.

**Figure 6:**
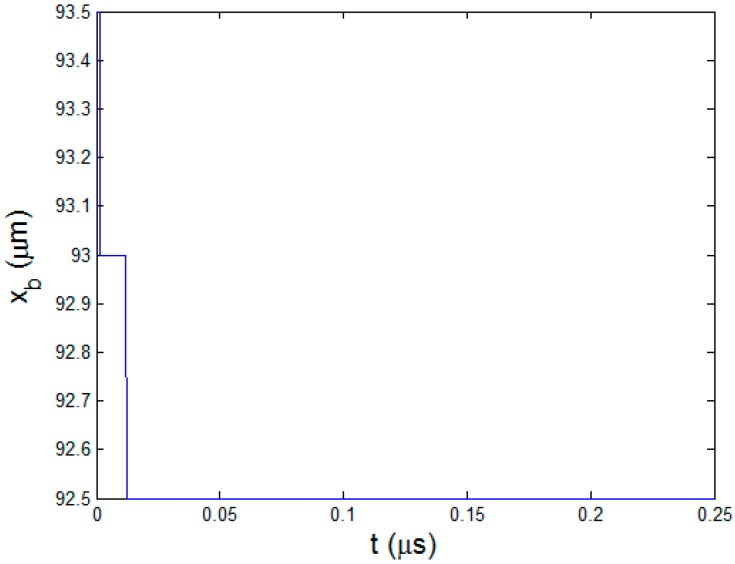
The curve of pyrolysis boundary $x_{b}$ at the cooling stage.

**Figure 7:**
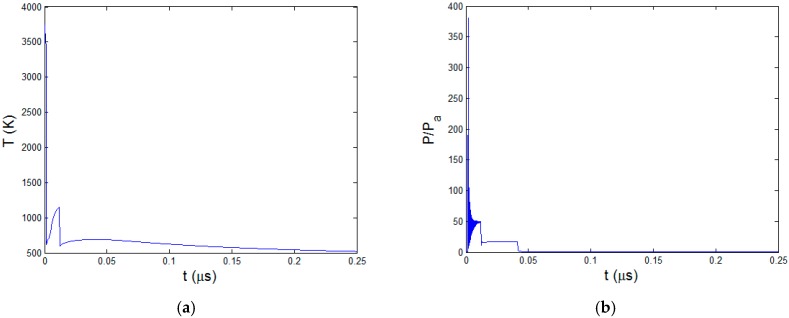
At the pyrolysis boundary $x_{b}$ (**a**) Temperature curve; (**b**) Pressure curve.

The ablation stops but the polymer pyrolysis continues with thermal diffusion. Figure 6 is the curve of pyrolysis boundary $x_{b}$ at the cooling stage. After the material cools down, the coordinate of $x_{b}$ does not change anymore. In this process, $x_{b}$ moves from 93.5 to 92.5 μm, forming 1 μm HAZ. The temperature curve and pressure curve at $x_{b}$ are shown in Figure 7. The temperature of the pyrolysis boundary rises because of the heating effect of ablation boundary. The material below is also heated up and becomes the new pyrolysis boundary when it reaches the degradation temperature. Therefore, the temperature curve in Figure 7a shows cyclical rise and decrease when the pyrolysis boundary moves. It finally converges to the polymer degradation temperature 593 K. After about 0.05 μs, temperature of the pyrolysis boundary starts to decrease and the pyrolysis process stops. In the pressure curve as shown in Figure 7b, it can be seen that the maximum pressure is more than 300 times the atmospheric pressure. In fact, this can be big enough to eject the exposed carbon fibers causing mechanical erosion of CFRP material which is experimentally confirmed in our previous research [8] and other research [6,17]. As the pyrolysis becomes slower, the pressure gradually decreases. Once the pyrolysis finishes, the pressure drops quickly to the atmospheric pressure due to the convection of pyrolysis gas.

In the laser milling experiments, the scanning speed, and hatching distance are set to be 1250 mm/s, and 0.25 mm, respectively. As the pulses repetition rate is 50 kHz, the interval between two adjacent laser pulses is about 0.25 mm which is close to the spot diameter. The power density within the focal laser spot has Gaussian distribution which is depicted as:

(15) $$I=\frac{2P}{\pi r^{2}}e^{-\frac{2\cdot (x^{2}+y^{2})}{r^{2}}}$$

**Figure 8:**
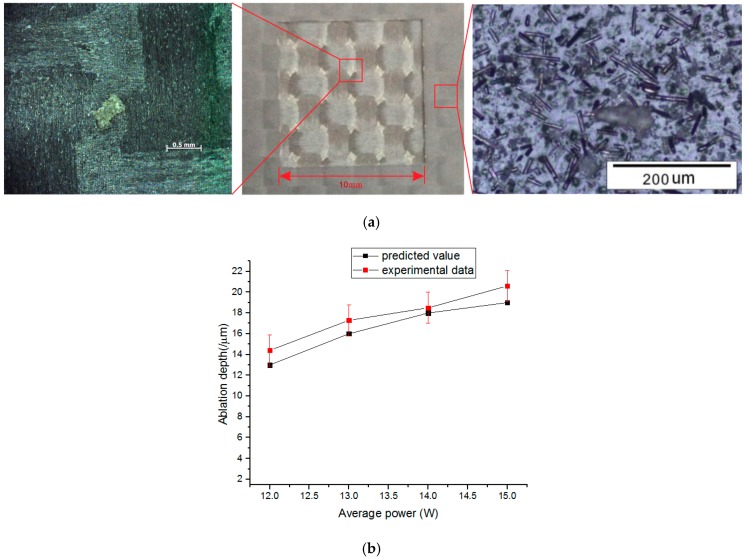
(**a**) Laser milling CFRP sample; (**b**) Predicted ablation depth vs. experimental data at different laser powers.

Due to the Gaussian distribution, the ablation dimple of a single pulse has a conical shape. For one scanning pass, one layer of material is removed. Figure 8a shows the milling sample at laser average power 12 W. It can be found that under the set pulses interval, chopped fibers between adjacent ablation dimples could be ejected by the high internal pressure and deposit around the machining area as shown in the right chart of Figure 8a. As the milling depth is less than the first ply, the woven texture can be clearly seen. The whole milling process displays uniform material removal feature generating a relatively flat bottom surface as shown in the left chart of Figure 8a. No obvious heat affected area is found. The laser average power varied from 12 to 15 W. For every milling sample, eight passes are repeated. The average depth of a single pass is calculated and compared with the numerical data. As the power intensity at the center of the laser spot is two times of the average power intensity used in the numerical model. The predicted value is doubled to perform the comparison. It is shown in Figure 8b that predicted ablation depths at different power levels conform well to the experimental results.

### 4.2. Effect of Laser Pulse Length

**Figure 9:**
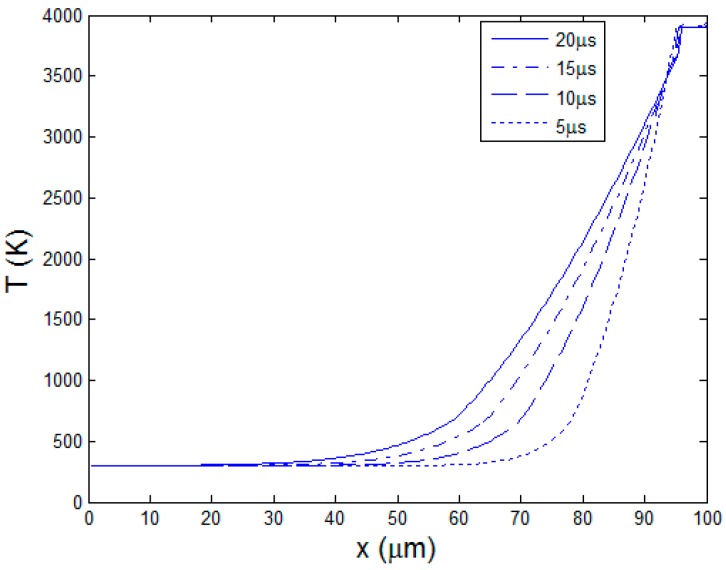
Temperature curves at the end of different pulse lengths.

**Figure 10:**
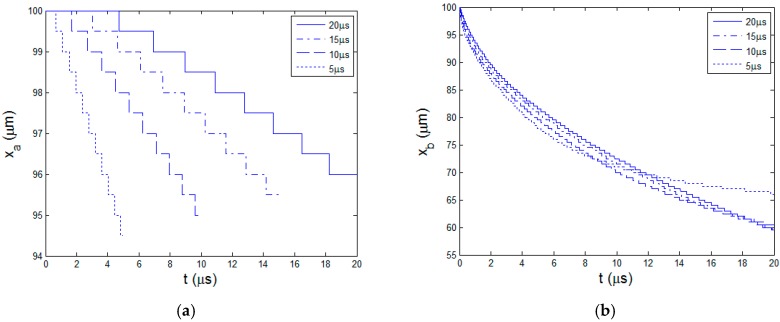
Moving boundaries at pulse lengths 5, 10, 15, 20 μs (**a**) Ablation boundary $x_{a}$; (**b**) Pyrolysis boundary $x_{b}$.

When the pulse duration increases, the material response is very different. Besides the 10 ns short pulse, other four pulse lengths 5, 10, 15, and 20 μs corresponding to the duty cycles, 25%, 50%, 75%, and 100% ( i.e. continuous wave mode, CW ), are studied. As the laser average power is kept constant, laser peak power intensity is inversely proportional to the laser pulse length. The four peak powers are 48, 36, 24, and 12 W, respectively which are much smaller than 2.4 × 10^4^ W of the 10 ns pulse length. Temperature curves at the end of the heating stages are shown in Figure 9. Different from the situation with short pulse length, the temperature around the ablation boundary $x_{b}$ decreases gradually to the initial temperature. Longer pulse length results in smaller temperature gradient in the material. The reason is that, at longer pulse length, the peak power is small and there is more time for the heat diffusion. Variation curves of the two boundaries $x_{a}$ and $x_{b}$ are drawn in Figure 10. As the peak powers are lower, the material needs a preheating time before reaching the ablation temperature under long pulses of μs order. At 5 μs, the preheating time is about 0.5 μs. At 20 μs, it is increased to 5 μs. After that, the ablation process proceeds continuously until the end of heating stage. However, the pyrolysis boundary curves in Figure 10b shows that the pyrolysis almost starts from the beginning and continues in the total pulse cycle. The material is easily heated to the polymer degradation temperature. It also indicates that at the cooling stage, temperature in the material does not completely fall below the polymer degradation temperature, producing large HAZ. With the enlargement of HAZ, the receding speed of pyrolysis boundary $x_{b}$ gradually decreases with time.

**Figure 11:**
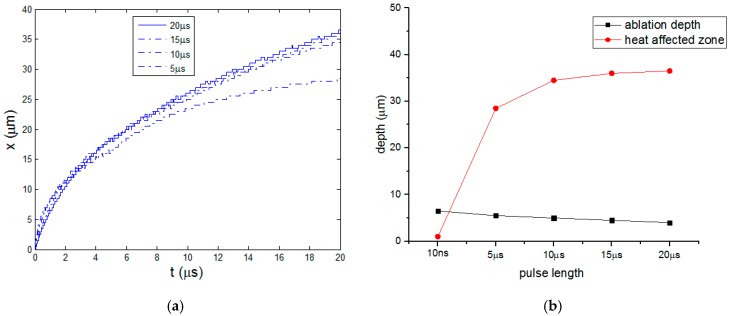
(**a**) Curves of HAZ; (**b**) Ablation depth and HAZ.

Figure 11a shows the curves of HAZ for the four pulse lengths. The increasing speeds of HAZ all decrease. When the pulse length is 5 μs, the size of HAZ almost stops increasing eventually as the cooling time is long. Figure 11b gives the comparison of ablation depth and heat affect zone at different pulse lengths. From 10 ns to 20 μs, the ablation depth decreases from 6.5 to 4 μm by 38.5%, however, the HAZ enlarges remarkably to about 36.5 μm which is nine times the ablation depth. At longer laser pulse more energy is not completely used to ablate the material but rather diffused to the original material. Then the energy utilization efficiency obviously decreases and material processing quality deteriorates. Because the phase change latency of polymer is far smaller than carbon fiber, the increasing amplitude of HAZ is much larger that the decreasing of ablation depth. The pyrolysis does not stop at the end of cooling stage for long pulse length in μs order. Polymer pyrolysis process would continue with the impact of next laser pulse and enlarges the HAZ further.

**Figure 12:**
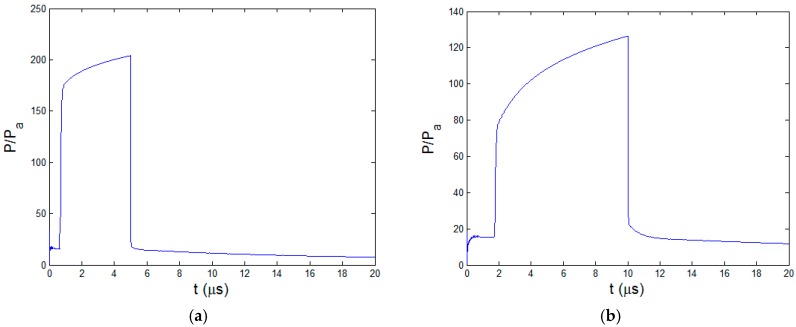

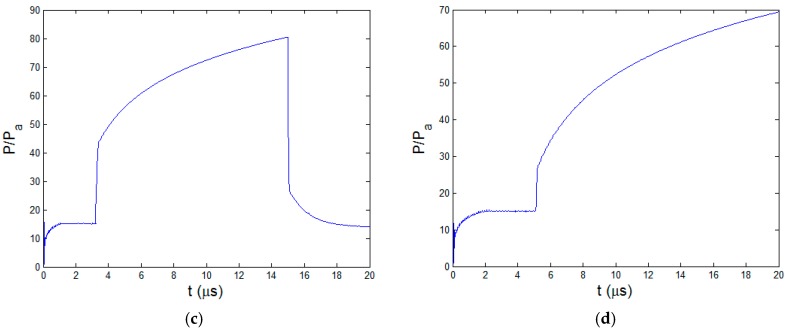
Pressure fields of different pulse lengths at $x_{b}$ (**a**) 5 μs; (**b**) 10 μs; (**c**) 15 μs; (**d**) 20 μs.

The time dependent pressures of different pulse lengths at the pyrolysis boundary $x_{b}$ are depicted in Figure 12. With longer laser pulse, the maximum pressure at $x_{b}$ decreases obviously, especially compared with the 10 ns laser pulse. At 5μs the maximum pressure is about 200 times $P_{a}$. It is just 70 times $P_{a}$ at 20 μs. At the start of the heating stage, material is heated to the polymer degradation temperature and a relatively large initial pressure is generated. Along with the extension of HAZ, the pressure increases and then reaches a constant value. When the material ablation starts, the ablation boundary is kept at ablation temperature. Meanwhile, enlargement of HAZ make the pyrolysis gas more difficult to flow outward. Therefore, the pressure increases until it reaches the maximum value at the end of the heating stage. At the cooling stage, the pressure drops quickly due to the gas filtration and the slowing down of pyrolysis rate. For the CW mode at 20 μs pulse length, as the power intensity is small compared to the other three cases, the initial pressure is a litter lower. However, because there is no cooling stage, the pressure continues to increase and reaches the maximum value at 20 μs. In a conclusion, the historic maximum pressure and pressure fluctuation increase when using shorter laser pulse length. This is beneficial to the utilization of gas mechanical erosion in the material removal.

## 5. Conclusions

Short pulsed laser processing has extremely high heating and cooling rate, especially in the polymer material whose heat conductivity is very small. Both the CFRP laser ablation and polymer pyrolysis behavior are studied in the established numerical model. Through the study of laser pulse length effect, following conclusions can be drawn:

(a) The material could be completely cools down and then polymer pyrolysis stops at the cooling stage at nanosecond pulse length. However, when the pulse length increases to μs order, the polymer pyrolysis continues even after the cooling stage. It generates multi-pulse heat concentration phenomenon enlarging the HAZ.

(b) It could be found that ablation depth is increased and HAZ is greatly reduced at short pulse length compared to long pulse length. Therefore, the shorter the pulse duration is, the bigger the material removal efficiency is. At nanosecond pulse length, the predicted ablation depth conforms well to the experimental data.

(c) The maximum historic pressure is inversely proportional to the pulse length. At laser pulse length in ns order, the internal pressure could reach over hundreds of times atmospheric pressure which can directly eject the exposed carbon fibers. This mechanical erosion phenomenon significantly increases the material removal efficiency.

## Figures and Tables

**Table 1:** Laser processing parameters and material properties of CFRP.

Laser processing parameters	Values	Material properties [8]	Carbon fiber	Epoxy matrix
Laser power *P* (W)	12	Volume fraction *φ*	0.6	0.4
Repetition rate *F* (kHz)	50	Density ρ (kg/m^3^)	1,850	1,200
Initial temperature *T*_0_ (K)	300	Heat conductivity *k* (W/(m·K))	50	0.1
Laser spot diameter (μm)	25	Heat capacity *C* (J/(kg^3^·K))	710	1,884
Absorptivity A	0.94 [14]	Latent heat *L* (kJ/kg)	43,000	1,000
		Degradation temperature *T*_1_ (K)	3,600	593
		Degradation temperature *T*_2_ (K)	3,900	773

**Table 2:** Material properties of pyrolysis gas.

Material properties	Values
Density ρ_g_ (Kg/m^3^)	1.16
Dynamic viscosity μ (Pa∙s)	3 × 10^−5^
Heat conductivity k_g_ (W/m∙K)	0.01
Heat capacity C (J/Kg∙K)	1,000
